# Drawing-Room Meeting in Edinburgh

**Published:** 1918-05-25

**Authors:** 


### THE NATION'S FUND FOR NURSES.

### Drawing-Room Meeting in Edinburgh.

WHILE thousands of nurses are working on the battlefields of France and Flanders, and ministering to our armies in Asia and Africa, there is something inspiring in the thought that those at home are taking up their cause, and striving to organise the nursing profession that it may be for ever worthy of its most heroic members. The work of the British Women's Hospital Committee in raising the Nation's Fund for Nurses has, until now, been little known in Scotland, and a meeting was, therefore, organised in Edinburgh on Wednesday, May 15, to set forth the objects of the Fund. Sir John and Lady Findlay of Aberlour kindly lent their house, and Lady Findlay presided over a representative audience.

The **Viscountess COWDRAY,** Hon. Treasurer of the Fund, who had come specially from London, spoke in glowing terms of the great work nurses had done in the past, and of how much all individually owed to them. She emphasised the necessity for the organisation of the profession, and for raising and unifying its educational standards. Up to the present nurses had not been able to get a Registration Bill passed. Even with the franchise many nurses would not have the vote. Everywhere they were realising the need for organisation, and nurses throughout the Dominions were showing great interest in the College. The British Women's Hospital Committee had called their fund, whose dual purpose was to endow the College, and to provide for aged and invalided nurses, the Nation's Fund, because it included not only Great Britain and Ireland, but the whole Empire. There would be vast changes after the war, and it was for those who were left at home to see that, so far as nurses were concerned, the changes were not for the worse.

The College had been built up during the war, largely with a view to the future of the profession, and already over 8,000 nurses were enrolled in it. This formed the largest Register of nurses that had ever existed, and they hoped it would grow; but until there was State Registration of every trained nurse, it was impossible to say even approximately how many trained nurses there were in the country. Lady Cowdray then appealed for money to help nurses broken down in health. She showed how pensions were only given by Government to nurses in the Services, how they took long to come, and how at best they only amounted to about £1 a week; in view of all that nurses had done she pleaded that, whatever happened, they should not be left to want. As they had little chance of saving, and a short working life, the future must be made sure for nurses if we were to get the right type of woman to enter the profession and maintain its high standards.

### THE ONLY SKILLED PROFESSION NOT ORGANISED.

**Miss GILL,** THE LADY SUPERINTENDENT of the Royal Infirmary of Edinburgh, was next called upon to speak. She said that nursing was probably the only skilled profession that was not organised. It had grown up in a chaotic fashion: there was no central representative authority, and nurses on leaving their hospitals were not responsible to anyone for their professional conduct. To begin with there were only a few training-schools: now hospitals of all kinds undertook to "train" nurses, and their training often fell far short of what it should be. There was no curriculum either for practical experience or for teaching, and there were no tests of the efficiency of nurses. But for the fact that the best hospitals trained their nurses highly, and that their efficiency reflected to the benefit of all, nurses as a class would have little claim at present to be called a skilled profession. For many years State Registration had been looked upon by ever increasing numbers as the remedy for this disorganised state of affairs, and much had been done to educate public opinion in the matter. Associations had been formed, and Bills promoted, but up to the end of 1915 there seemed to be no chance of legislation. Miss Gill then went on to show how the war had emphasised the need of organisation, and how the College had come into being as an outcome of the nurses' desire to organise. She paid a high tribute to the splendid services Sir Arthur Stanley had rendered to the whole profession in his pioneer work in connection with the College of Nursing, and outlined the work of the College in connection with the Register, the preparation of a Bill for State Registration, and nursing matters generally.

### SOME FINANCIAL QUESTIONS.

After showing what were the aims of the College, and giving some account of the constitution and *personnel* of the Scottish Board, Miss Gill turned to the question of finance, and pointed out that without an endowment it would be impossible for the College of Nursing to realise its ideals. In past generations public and educational institutions had been endowed by the generosity of beneficent founders, and there could be no better object to-day than the establishment of the nursing profession on a sound and lasting basis. If, through want of organisation and bad conditions, we were unable to attract the best type of woman, it would be a great loss to the country at large; and there was a very decided danger that, if something were not done soon, nursing would cease to appeal to educated women, and the profession would have to be recruited from the leavings. We all looked for the highest qualities in nurses, but only the matrons of large institutions knew how difficult it was to get good material to train. We stood on the threshold of a new era for women, and the nursing profession, if it were to maintain the highest ideals and traditions of the past, would have to move forward.

**Mrs. ALDERTON,** OF COLCHESTER, explained how the committee which had raised the great Star and Garter Fund was about to dissolve when Sir Arthur Stanley asked them to undertake the task of collecting money for the endowment of the College of Nursing. She had not been connected with the committee in those days, so she was not boasting when she said they had raised a maximum amount with a minimum expenditure, and she was confident that such a committee would succeed in raising the sum required for the Nation's Fund for Nurses. They had learned to look at the whole matter from the nurse's point of view. The profession badly needed organisation, and this could only be perfected by spending money. It was an interesting, but an extraordinarily arduous, profession, and a nurse must maintain her health, her spirits, and her soul at a very high level. It was difficult to do this without organisation in the profession, and with long hours and poor pay. By helping the College of Nursing to organise the committee was doing good, not only to nurses, but also to the public. With regard to recruits, Mrs. Alderton said that over and over again high-school girls said to her that they would like to go in for nursing, but that their parents were unwilling that they should do so as the prospects were so poor. We must make the nursing profession very different from what it was if we would attract girls leaving school and taking college or high-grade qualifications. One and all of them should choose a career. They knew that for tragic reasons many of them would remain unmarried, and that their professional work must render them economically independent. So the question of a nurse's pay became a very important one.

**Mrs. GEORGE KERR** said that the other speakers had left little for her to add, but that she would like to point out that the response of the nursing profession to the call of the nation had never been affected by difficulties or wages. Nurses had never been deterred by these things. But, in the more commonplace days after the war, would it be possible for them all to keep such high standards before them? Surely it would be easier for nurses, if they belonged to the College, a great union whose intention was that its members should work with high ideals, to go forward as a body on the great mission that was set before them.

The **Rev. JOHN KELMAN**, who had recently returned from visiting the troops in France and Italy, in proposing a vote of thanks to Lady Cowdray and the other speakers, paid a high tribute to the work of nurses in the war zone. He recalled many incidents of the worth of a woman's presence to suffering soldiers in their hour of need, and some notable examples of nurses' heroism. On the motion of the Very Rev. Dean Skinner Wilson a hearty vote of thanks was accorded to Sir John and Lady Findlay. £49 2s. was given at the meeting.

